# In vivo study of newly developed albumin-conjugated urate oxidase for gout treatment

**DOI:** 10.1186/s13075-023-03231-3

**Published:** 2023-12-18

**Authors:** Jeonghaeng Cho, Byungseop Yang, Jae Hun Lee, Hyunwoo Kim, Hyeongseok Kim, Eun Byeol Go, Dong-ho Bak, Su Jin Park, Inchan Kwon, Jong-il Choi, Kyunghee Lee

**Affiliations:** 1 Research and Development, ProAbTech Co., Ltd, Seoul, 07807 Republic of Korea; 2 Department of Biotechnology and Bioengineering, Interdisciplinary Program for Bioenergy and Biomaterials, Chonnam National University, Gwangju, 61186 Republic of Korea; 3 School of Materials Science and Engineering, Gwangju Institute of Science and Technology (GIST), Gwangju, 61005 Republic of Korea

**Keywords:** Gout, Urate oxidase, *Af*Uox, rHA, Site-specific conjugation, IEDDA, frTet, PAT101

## Abstract

**Background:**

Exogenously providing engineered Uox with enhanced half-life is one of the important urate-lowering treatments for gout. The potential of PAT101, a recombinant human albumin (rHA)-conjugated variant, was evaluated and compared as a novel gout treatment through various in vivo studies with PAT101 and competing drugs.

**Methods:**

PAT101 was produced by site-specific conjugation of rHA and *Aspergillus flavus* Uox (*Af*Uox-rHA) through clickable non-natural amino acid (frTet) and Inverse electron demand Diels–Alder (IEDDA) reaction. In vivo pharmacokinetics, efficacy tests and in vitro immunogenetic assay were performed after single or multiple doses of PAT101 and its competitors in BALB/c mice, transgenic (TG) mice, Sprague–Dawley** (**SD) rats, and non-human primate (NHP).

**Results:**

The half-life of PAT101 in single-dose treated TG mice was more than doubled compared to pegloticase. In SD rats with 4 weeks of repeated administration of rasburicase, only 24% of Uox activity remained, whereas in PAT101, it was maintained by 86%. In the Uox KO model, the survival rate of PAT101 was comparable to that of pegloticase. In addition, human PBMC-based CD4^+^/CD8^+^ T-cell activation analysis demonstrated that PAT101 has a lower immune response compared to the original drug, rasburicase.

**Conclusion:**

All results suggest that this rHA-conjugated *Af*Uox, PAT101, can be provided as a reliable source of Uox for gout treatment.

**Supplementary Information:**

The online version contains supplementary material available at 10.1186/s13075-023-03231-3.

## Background

Gout is an inflammatory arthritis defined as uric acid levels higher than 6.8 mg/dL, resulting in the formation of monosodium urate crystals that trigger an acute inflammatory response. This eventually leads to chronic inflammation due to uric acid accumulations in joints and other tissues [1–3]. Urate oxidase (Uox) is a homo-tetrameric enzyme composed of four identical 34 kDa subunits that catalyze the conversion of insoluble uric acid to the water-soluble 5-hydroxyisourate and allantoin following a series of reactions [4]. In higher primates including humans, a functional Uox enzyme is not expressed due to three mutations that result in the complete silencing of the gene [5, 6]. Therefore, intravenous administration of Uox can be used as an enzyme therapy for gout by supplying enzymes lost during hominoid evolution in humans [3]. Allopurinol is the most used therapeutic drug, being used by more than 2 million gout patients in the US. However, despite this treatment, many high-risk patients still have higher than acceptable levels of uric acid [7]. Of the various enzyme therapy developments to date, only two engineered uricases, rasbricase (Fasturtec®) and pegloticase (Krystexxa®), are clinically approved [8, 9]. Rasburicase is a recombinant Uox derived from *Aspergillus flavus* (*Af*Uox) and has been demonstrated to be therapeutically superior to allopurinol in regulating serum urate levels in adult patients [10, 11]. However, *Af*UOX has poor stability and short plasma half-life. To solve this problem, a PEGylated Uox variant was developed. Pegloticase is a PEGylated chimeric porcine–baboon Uox developed to extend the serum half-life of therapeutic Uox in vivo [12–14]. Nevertheless, several concerns regarding pegloticase have been raised about the immunogenicity and toxicity of PEG (polyethylene glycol)-conjugated molecules, such as generating antibodies against PEG and the low degradability of PEG molecules. Moreover, heterogenous PEGylation and ADA (anti-drug antibody) formation hinder the functional activity of Uox. Consequently, the development of alternative Uox-based therapeutic agents to mitigate these concerns is still in high demand.

It has been well-known that albumin has little immunogenicity and is biodegradable unlike PEGs. It also has an extremely long intrinsic serum half-life (over 3 weeks) via the neonatal Fc receptor (FcRn)-mediated recycling mechanism [15–18]. Based on these reasons, albumin could be proposed as a therapeutic carrier that can replace the immunogenicity-inducing PEGs, while simultaneously maintaining the advantage of increasing the in vivo half-life of Uox for this study. Previous studies have already confirmed that site-specific albumin conjugation following the genetic incorporation of a non-natural amino acid (NNAA) prolonged the therapeutic activity of Uox in vivo [19]. Meanwhile, the conjugations of recombinant human albumin (rHA) to tetrameric Uox for increased purity, that is, in the form of three or four albumin conjugation, had to be improved. In the development process, the inverse electron demand Diels − Alder reaction (IEDDA), a bio-orthogonal chemistry due to its faster reaction kinetics compared to strain-promoted azide-alkyne cycloaddition (SPAAC), has been adapted to improve the purity [20]. In addition, it was studied that the incorporation of phenylalanine analog containing hydrogen-substituted tetrazine (frTet) into a specific site of the Uox was effective in increasing the generation of three or four albumin-conjugated Uox variants through IEDDA reaction [20]. Finally, we have developed a rHA-conjugated Uox with high clinical applicability by investigating a site-specific conjugation of rHA to *Af*Uox and designated this conjugated biomolecule as PAT101.

Here, we investigated the characteristics of PAT101 as a therapeutic Uox as follows: in vivo extended serum half-life profiles in mice and rats, in vivo therapeutic efficacy through plasma uric acid reduction in chronic Uox-KO mice model, and potential for low immunogenicity as measured in a T-cell immunogenicity assay. Results have shown that the conjugation of rHA to a selective site of *Af*Uox leads to an extended half-life through an FcRn-mediated recycling system as well as a volume-induced renal filtration evasion mechanism. Above all, the low immunogenicity compared to rasburicase suggests that PAT101 is a promising gout treatment that overcomes several issues reported in previous treatments.

## Methods

### In vitro production and purification of PAT101

The production of PAT101 involved a process flow with upstream and downstream procedures. In the upstream phase, the cell stock C321delA.exp.[pDule C11RS][pTAC *Af*Uox 174 Amb] was cultivated in 2xYT media supplemented with kanamycin (35 μg/mL) and tetracycline (10 μg/mL). The seed culture was scaled up to a 5 L fed-batch culture with the addition of 1 mM IPTG and 3 mM frTet for induction. After 14 hrs, cells were harvested by centrifugation at 8000 rpm for 15 min at 4 °C. In the downstream phase, the cell pellet was lysed using a high-pressure microfluidizer in a 20 mM sodium phosphate buffer (SPB) pH 7.0. The lysate was purified using cation exchange chromatography. The purified *Af*Uox-174 frTet was used for recombinant human albumin conjugation, where albumin reacted with a crosslinker (TCO-MAL) for 2 hrs, followed by crosslinker removal through diafiltration. Uox-174frTet and rHA-TCO were conjugated at a 1:6 molar ratio in a SP pH 7.0 for 1 h. After conjugation, the mixture was desalted and subjected to two-step chromatography purification. First, cation exchange chromatography (SP Sepharose FF) was performed with SPB pH 6.0. Then, anion exchange chromatography (Q Sepharose FF) was carried out with SPB pH 7.0, resulting in the purified PAT101.

### Animals

Experiments were performed in accordance with the guidelines on the Care and Use of Laboratory Animals after approval from the Institutional Animal Care and Use the Committees of Kbio Health (KBIO-IACUC), Gwangju Institute of Science and Technology (GIST) and Ajou University (AJOU). The approval number was specified for each study.

### Single Dose Pharmacokinetic (PK) studies in BALB/c (GIST-2021–092) and TG mice (KBIO-IACUC-2021–207)

Six-week-old female BALB/c mice (*n* = 5–6 per group) were intravenously administrated with rasburicase (Sanofi, France), pegloticase (Savient Pharm. NJ, USA), and PAT101 (ProAbTech Co., Ltd., S. Korea) at a dosage of 2.0, 2.0, and 6 mg/kg b.w./day, respectively. Blood samples were collected at different time points: 0.25, 2, 4, and 8 hr for rasburicase, and 0.25, 2, 4, 8, 12, 24, 36, and 48 hr for both pegloticase and PAT101. Another single-dose PK study of PAT101 and pegloticase was performed using TG mice (strain: B6. Cg-Tg (FCGRT)32Dcr Albem12Mvw Fcgrttm1Dcr/MvwJ) generated at Jackson Laboratory (USA) (n = 10 per group). The blood was collected at the scheduled timepoint, 0, 0.5, 1, 2, 4, 8, 24, 48, 96, 168, 240, and 336 hr following a single I.V. injection of either PAT101 (6.0 mg/kg) or pegloticase (2.0 mg/kg).

### Repeated-dose 4-week PK study using Sprague–Dawley (SD) rats (KBIO-IACUC-2021–206)

Either PAT101 (1.0 mg/kg) or rasburicase (0.325 mg/kg) was administrated into the caudal vein of 6-week-old male SD rats (KOATECH, Korea) (*n* = 5 per group) once a week for a total of 4 times and blood was collected at a scheduled time point.

### Generation of Uox knockout (KO) mice (AJOU-2016–0041)

C57BL/6 J-background Uox heterozygous KO mice (*Uox*^+/−^) (B6;129S7-Uoxtm1Bay/J/Strain #002223) were purchased at Jackson Laboratory (https://www.jax.org/strain/002223) (Maine, USA). After mating with Uox hetero mice, Allopurinol (25 mg/kg) (Yuyu Pharma, Inc., S. Korea) was administered daily to pregnant female mice through oral administration. 14 days after giving birth, genotype was performed on all the Uox KO mice and Uox homozygous KO (− / −) was selected. PAT101 or pegloticase was administered to Uox homozygous KO (− / −) mice every week through intraperitoneal injection (I.P.). Test substances and each dosage are shown in Table 1. The Uox homozygous KO mice line was maintained as described previously [21].

**Table 1:** The information of test substances, dosage, and administration interval by groups

Group	# of mice	Test substance	Route of administration	Dosage (mg/kg)	Interval
G1	5	KO	-	-	-
G2	5	KO + allopurinol	Allopurinol (oral)	25	Allopurinol (once daily)
G3	5	KO + allopurinol + PAT101	Allopurinol (oral) + PAT101 (I.P.)	Allopurinol (25) + PAT101 (6)	Allopurinol (once daily) + PAT101 (once weekly)
G4	5	KO + allopurinol + pegloticase	Allopurinol (oral) + pegloticase (I.P.)	Allopurinol (25) + pegloticase (2)	Allopurinol (once daily) + Pegloticase (once weekly)

### Genotyping

WT, and Uox KO (Uox.^−/−^) mice were identified by PCR amplification of genomic DNA isolated from toe tissue. Primers and PCR reactions followed those described previously [22]

### Histology and Histopathologic scoring

Histology of the kidney was evaluated on 2- to 3-μm sections of paraffin-embedded tissues stained with H&E. The histological score is determined according to the following criteria after confirming the degree of grading tubular necrosis, loss of brush border, cast formation, and tubular dilatation; score 0: none, score 1: < 10%, score 2: 10–25%, score 3: 25–50%, score 4: 50–74%, score 5: > 75%.

### Uric acid measurement in plasma

A blood sample was collected in a plasma separation tube (PST) and mixed lightly. Plasma was obtained by centrifugation at 4 °C at 6000 rpm for 10 min. The concentration of uric acid in plasma samples was measured spectrophotometrically (590 nm), using an enzymatic uric acid assay kit (cat. # KA1651, Abnova Taipei Taiwan), according to the manufacturer’s protocol.

### Pharmacokinetic analysis

After mixing the plasma and uric acid at 105.3 µM in assay buffer (7.5 g/L triethanolamine and 0.38 g/L EDTA at pH 8.9), it was loaded onto the heated UV-STAR® microplates at 30°C incubator for 10 min, and the absorbance change (initial rate) was measured using a microplate reader (293 nm). Enzyme activity in plasma was quantified as specific activity (mU/mL), where one unit (mU) of activity indicated the quantity of enzyme catalyzing the oxidation of 1.0 nmol of uric acid per minute at 30 °C. Plasma pharmacokinetic parameters were calculated using non-compartmental analysis (NCA) with WinNonlin software (version 8.1.0, Pharsight Corporation, Mountain View, California) or PK solver [23].

### Peripheral blood mononuclear cells (PBMCs)-based assay in vitro

The potential immunogenicity of PAT101 was assessed in human PBMCs based on a previous report with appropriate modifications [24, 25]. Immunogenicity was expressed as stimulation index (SI) as follows: simulation index = sample well/baseline.

### Statistical analyses

Statistical analysis in uric acid level and histological score of Uox KO mice study was performed using one-way ANOVA (by a Bonferroni correction for multiple comparisons) and survival rate was done using the Mantel–Haenszel method. All analyses were performed using Prism 5.0 (GraphPad Software, Inc., CA, USA). Data is expressed as mean ± SEM. In all statistical analyses, *p* < 0.05 was considered statistically significant.

## Results

### Single Dose PK studies

**Fig. 1:**
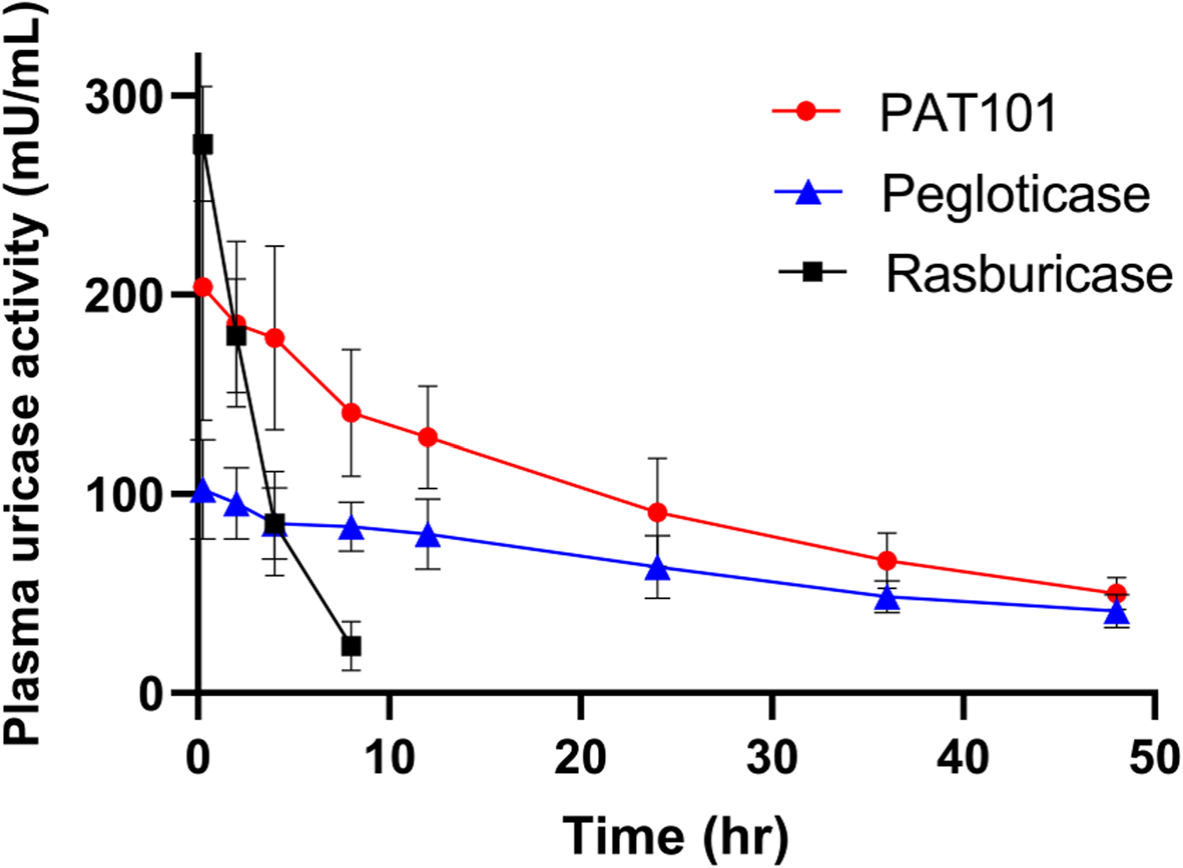
Comparative single dose PK profiles of PAT101, pegloticase, and rasburicase in BALB/c mice. PAT101, pegloticase, and rasburicase were injected to female BALB/c mice (*n* = 5 or 6). The injected test substances have the same molar concentration of uricase. The blood samples were collected at different time points: 0.25, 2, 4, and 8 hr for rasburicase, and 0.25, 2, 4, 8, 12, 24, 36, and 48 hr for pegloticase and PAT101. The calculated plasma uricase activity (mU/mL) in the log scale was plotted against time (hr) to examine the PK profiles. The values of PK parameters, half-life, and AUC are shown as mean ± SD

**Fig. 2:**
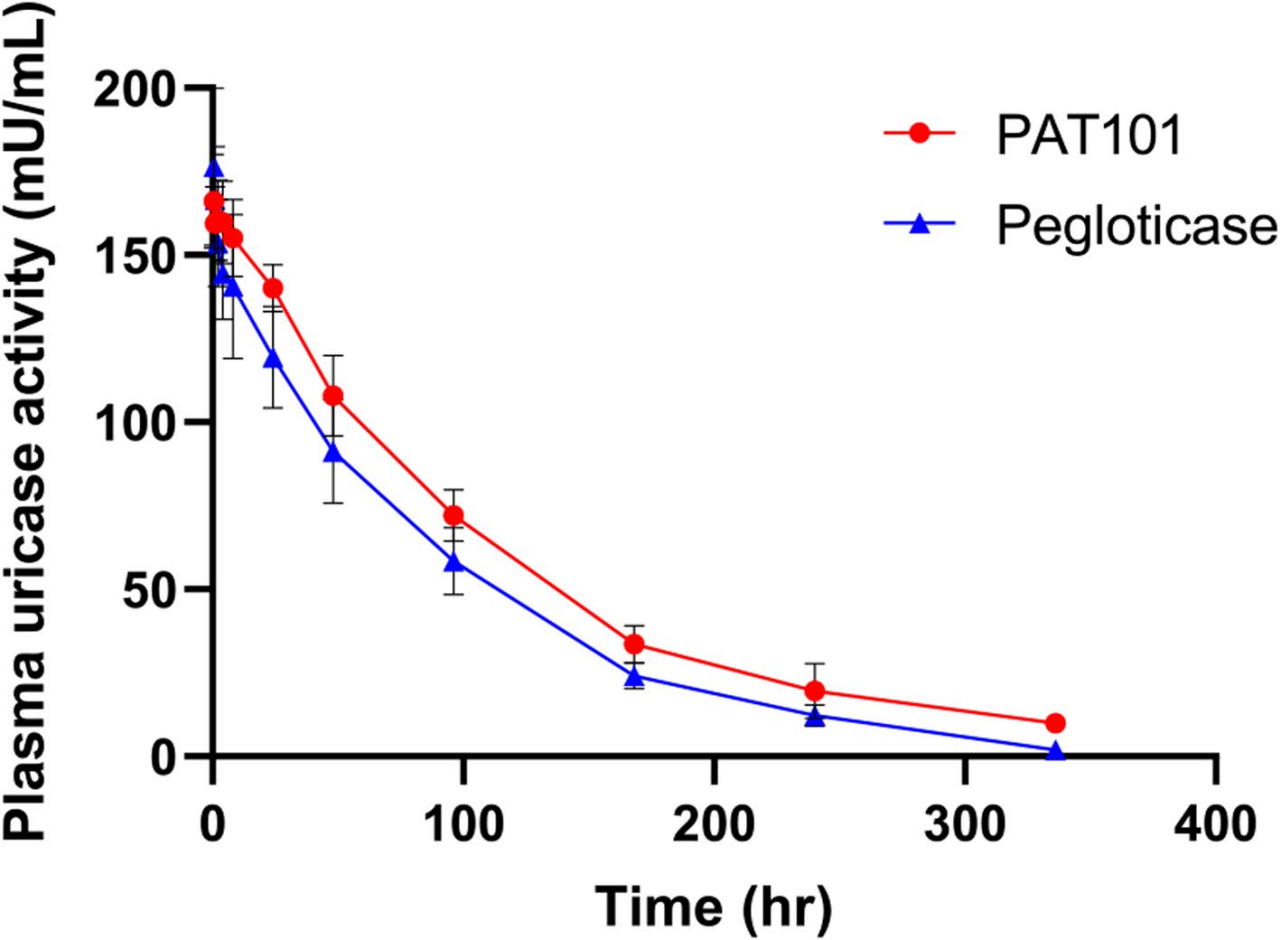
Comparative single dose PK profiles of PAT101 and pegloticase in TG mice. After intravenous injection of PAT101 (*n* = 5) or pegloticase (*n* = 5) into TG mice, the residual PAT101 and pegloticase in plasma were measured using enzymatic activity assay at indicated time points (0.5, 1, 2, 4, 8, 24, 48, 96, 168, 240, and 336 hr after administration). The uricase molar concentration of the injected test substances is the same. The calculated plasma uricase activity (mU/mL) in log scale was plotted against time (hr) to examine the PK profiles. The values of PK parameters, half-life and AUC, are shown as mean ± SD

In BALB/c, the measured plasma uricase activity (mU/mL) in linear log scale was plotted against time (hr) to examine the pharmacokinetic profiles of three Uox variants, rasburicase, pegloticase and PAT101 (Fig. 1). As shown in Fig. 1 and Table 2, compared to rasburicase, the half-lives (T_1/2_) of PAT101 and pegloticase were extended 15-fold and 20-fold, respectively. In addition, PAT101 showed a 30–40% higher AUC than pegloticase, indicating that PAT101 has better systemic exposure and clearance in the body. To project human PK of PAT101, a PK profile was also investigated in TG mice expressing human neonatal Fc receptor (hFcRn). Several pharmacokinetic parameters derived from non-compartmental analysis (NCA) of the 2 groups are summarized in Table 3. T_max_ (Time to reach C_max_) was the same and C_max_ (peak plasma concentration) was similar between groups. Above all, the half-life of PAT101 (95 h) was more than twice as long as that of pegloticase (45 h) in this model, confirming that PAT101 can extend the half-life and AUC of Uox in humans through FcRn-mediated albumin recycling mechanism (Fig. 2 and Table 3).

**Table 2:** Pharmacokinetic parameters using non-compartmental analysis

Parameters	Rasburicase (2.0 mg/kg)	Pegloticase (2.0 mg/kg)	PAT101 (6 mg/kg)
T_1/2_ (hr)	2.03 ± 0.48	38.59 ± 11.11	31.81 ± 11.31
AUC_last_ (h*mU/mL)	951.73 ± 169.99	3110.87 ± 550.31	4891.35 ± 1065.23

**Table 3:** Pharmacokinetic parameters using non-compartmental analysis

Parameter	PAT101 (1 mg/kg)	Pegloticase (2 mg/kg)
T_1/2_ (hr)	95.02 ± 12.56	45.23 ± 9.18
T_max_ (hr)	0.50 ± 0.00	0.50 ± 0.00
C_max_ (mU/mL)	166.05 ± 14.02	176.37 ± 23.60
AUC_last_ (h*mU/mL)	18,067.84 ± 1577.39	14,365.17 ± 1901.47

### Multiple Dose PK study in SD rats

**Fig. 3:**
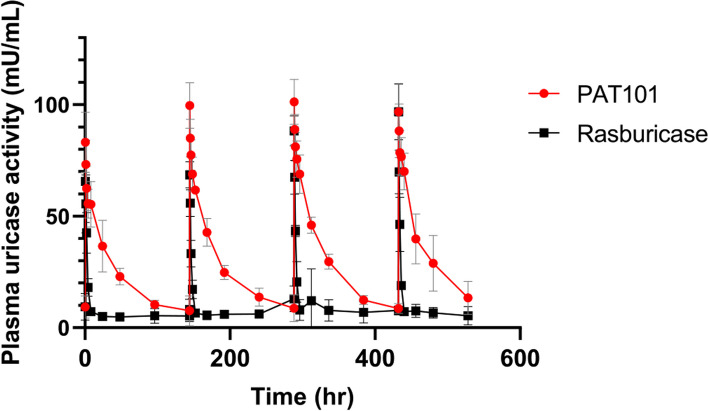
Comparative repeated dose PK profiles of PAT101 and rasburicase in SD rats. After weekly dosing of PAT101 or rasburicase for 4 weeks, plasma concentration versus time profiles were measured using enzyme activity assay at indicated time points (0.5, 1, 2, 4, and 8 hr after administration). Each test substance was injected at the same molar concentration of uricase. The calculated plasma uricase activity (mU/mL) in log scale was plotted against time (hr) to examine the PK profiles. Data are plotted as mean ± SD of 10 rats

The pharmacokinetic profile results after repeated administration for 4 weeks are shown in Fig. 3 and Table 4. After the 1st, 2nd, 3rd, and 4th administration, the C_max_ of PAT101 was 83.11, 99.65, 101.28, and 96.78 mU/mL, respectively, and that of rasburicase was confirmed to be 65.60, 68.47, 88.20, and 96.78 mU/mL, respectively. The half-life of PAT101 was 59.94, 45.63, 38.12, and 44.28 hr, respectively, with an average of 46.99 ± 9.23 hr (average ± SD), and that of rasburicase was 2.45, 2.71, 2.56, and 2.38 hr, respectively, with an average of 2.53 ± 0.14 hr (average ± SD). During repeated 4-week dosing, the half-life of PAT101 was, on average, about 20 times longer than that of rasburicase. Both showed the highest blood drug concentration at 0.5 hr but the AUC was about 10 to 12 times wider in PAT101 than in rasburicase after repeated dosing. PAT101 maintains a 86% activity even after repeated administration for 4 weeks, whereas the activity of the original drug rasburicase decreased to 24% after 4 weeks. Other pharmacokinetic parameters, T_max_, V_z_, and Cl were not different between the two substances. It was demonstrated that the activity of PAT101 unlike rasburicase was maintained even after repeated administration.

**Table 4:** Pharmacokinetic parameters using non-compartmental analysis

Parameter	After the first injection		After the second injection		After the third injection		After the fourth injection	
	**PAT101** **(1 mg/kg)**	**Rasburicase** **(0.325 mg/kg)**	**PAT101** **(1 mg/kg)**	**Rasburicase** **(0.325 mg/kg)**	**PAT101** **(1 mg/kg)**	**Rasburicase** **(0.325 mg/kg)**	**PAT101** **(1 mg/kg)**	**Rasburicase** **(0.325 mg/kg)**
T_1/2_ (hr)	59.94 ± 10.23	2.45 ± 0.34	45.63 ± 13.68	2.71 ± 0.64	38.12 ± 3.75	2.56 ± 0.94	44.28 ± 16.99	2.38 ± 0.46
T_max_ (hr)	0.50 ± 0.00	0.50 ± 0.00	0.50 ± 0.00	0.50 ± 0.00	0.50 ± 0.00	0.50 ± 0.00	0.50 ± 0.00	0.50 ± 0.00
C_max_ (mU/mL)	83.11 ± 13.51	65.60 ± 18.44	99.65 ± 10.20	68.47 ± 5.86	101.28 ± 10.17	88.20 ± 6.73	96.78 ± 12.64	96.78 ± 12.43
AUC_last_ (hr*mU/mL)	3167.12 ± 579.44	307.27 ± 86.70	3156.94 ± 203.78	211.76 ± 22.49	3467.72 ± 241.20	266.18 ± 39.36	3346.13 ± 908.90	274.83 ± 36.50
AUC_all_ (hr*mU/mL)	3167.12 ± 579.44	362.28 ± 100.81	3156.94 ± 203.78	264.44 ± 33.37	3467.72 ± 241.20	330.13 ± 75.26	3346.13 ± 908.90	332.11 ± 41.02
AUC_inf_ (hr*mU/mL)	3838.56 ± 688.02	324.51 ± 89.50	4112.70 ± 529.69	238.64 ± 31.18	4156.78 ± 349.05	300.61 ± 67.02	4330.31 ± 1591.65	300.03 ± 36.23
AUC_%Extrap_ (%)	17.30 ± 5.02	5.47 ± 0.94	22.35 ± 9.57	10.98 ± 4.28	16.46 ± 3.16	10.22 ± 7.46	19.68 ± 11.68	8.47 ± 3.40
V_z_ (mg/mU/mL)	0.00 ± 0.00	0.00 ± 0.00	0.00 ± 0.00	0.00 ± 0.00	0.00 ± 0.00	0.00 ± 0.00	0.00 ± 0.00	0.00 ± 0.00
Cl (mg/hr*mU/mL)	0.00 ± 0.00	0.00 ± 0.00	0.00 ± 0.00	0.00 ± 0.00	0.00 ± 0.00	0.00 ± 0.00	0.00 ± 0.00	0.00 ± 0.00

*T*_*1/2*_ half-life, *T*_*max*_ Time at maximal concentration, *C*_*max*_ Maximal concentration, *AUC*_*last*_ Area under the curve from administration to the last measured concentration, *AUC*_*all*_ Area under the curve from the time of dosing to the time of the last observation, *AUC*_*inf*_ Area under the curve from administration to infinity, *AUC%*_*Extrap*_ Percentage of the extrapolated area under the curve at the total area under the curve, *Vz* The volume of distribution, *Cl* Clearance, *Mean* Average, *SD* Standard deviation

### PAT101 efficacy study in Uox KO mice model

**Fig. 4:**
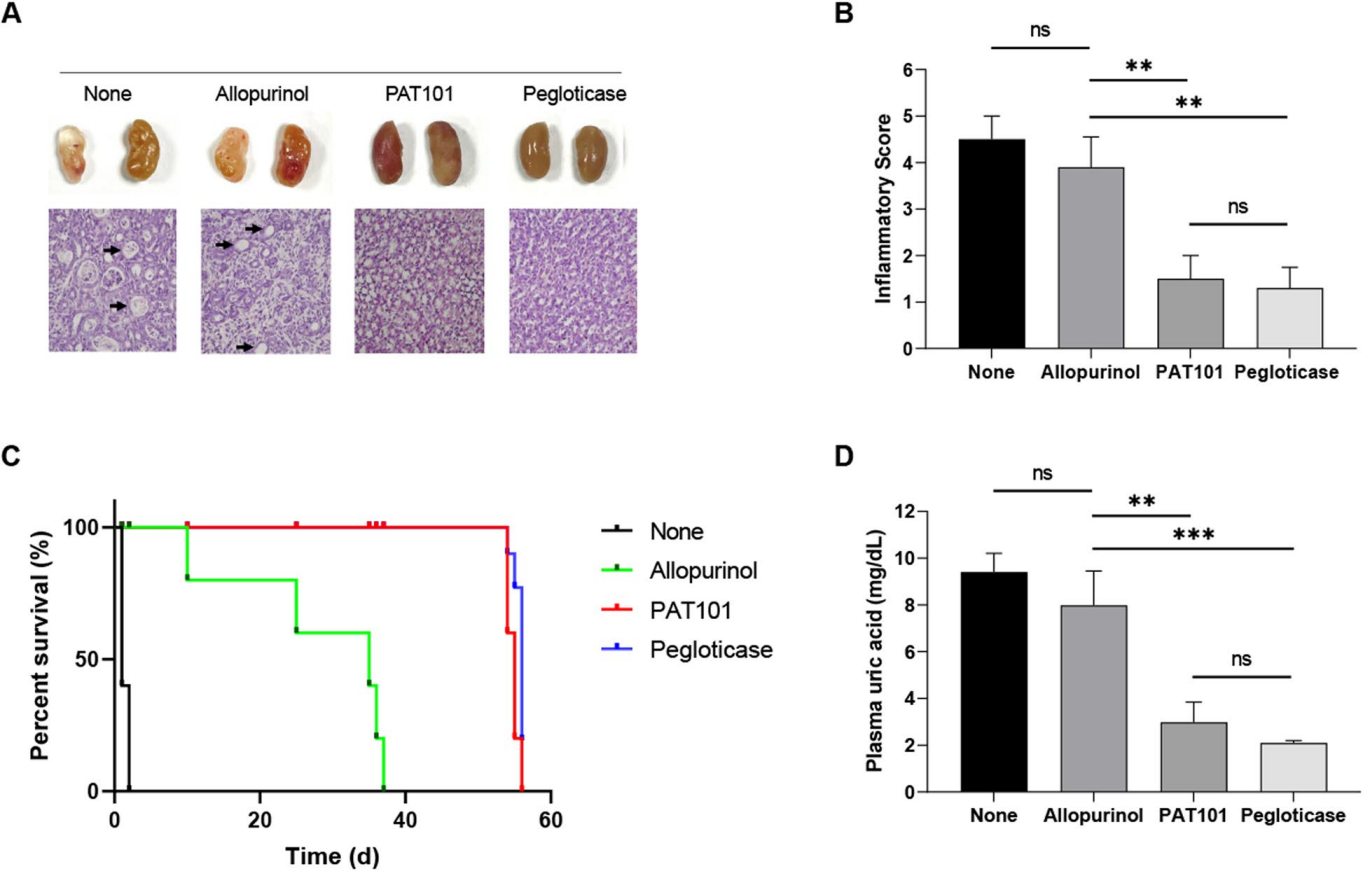
Efficacy of PAT101 and pegloticase on pathological kidneys in Uox KO mice. **A** Macroscopic evaluation (upper panel) and H&E staining results (lower panel) of Uox KO mouse kidney tissues untreated or treated with either allopurinol, PAT101, or pegloticase. Tubular damage with dilated tubules is indicated by black arrows. **B** The histological inflammatory score was evaluated by one-way analysis of variance (ANOVA) statistical analysis containing tubular necrosis, loss of brush border, tubular dilatation, and cellular infiltration. The scoring was evaluated following the score 0: none, score 1: < 10%, score 2: 10–25%, score 3: 25–50%, score 4: 50–74%, score 5: > 75%), *P* value (ns: not significant, **: < 0.01). **C** Kapan-Meier survival analysis in four Uox KO groups without or with 3 different treatments such as allopurinol, PAT101, or pegloticase. **D** Analysis of uric acid concentration in Uox KO mice in four Uox KO groups without or with three different treatments such as allopurinol, PAT101, or pegloticase. The residual plasma uric acid was measured by the uric acid assay kits (Abnova, Taipei Taiwan). ns: not significant, **: < 0.01, ***: < 0.001

Results from histological and morphological analysis of kidney tissues in untreated and Allopurinol-treated groups showed tubular necrosis and dilated tubules (Fig. 4A). However, no pathological findings were observed in both PAT101 and pegloticase treated Uox KO mice (Fig. 4A). The histological inflammatory score was evaluated according to the degree of tubular necrosis, loss of brush border, tubular dilatation, and cellular infiltration, indicating that PAT101 as well as pegloticase can restore the pathological inflammation in Uox KO mice (Fig. 4B). To estimate the survival rate from each treatment, Kaplan–Meier curves were used as shown in Fig. 4C. The overall survival of Uox KO mice was from Day10 to Day36 when Allopurinol was administered from Day1 after birth, However, animals survived up to Day56 in Uox KO mice when administered with PAT101 or pegloticase (Fig. 4C). If the body weight of each test group decreased by less than 30%, it was regarded as hyperuricemia, and uric acid level was measured after blood collection. The uric acid level of Uox KO mice was on average 9.4 ± 0.7 mg/dL. After administration of allopurinol, PAT101, pegloticase, and uric acid level was at 7.9 ± 1.5, 3.0 ± 0.9, and 2.1 ± 0.1 mg/dL, respectively. The treatment of PAT101 and pegloticase dramatically decreased the uric acid level in Uox KO mice (Fig. 4D).

### T cell subset counts for PAT101 immunogenicity assessment

**Fig. 5:**
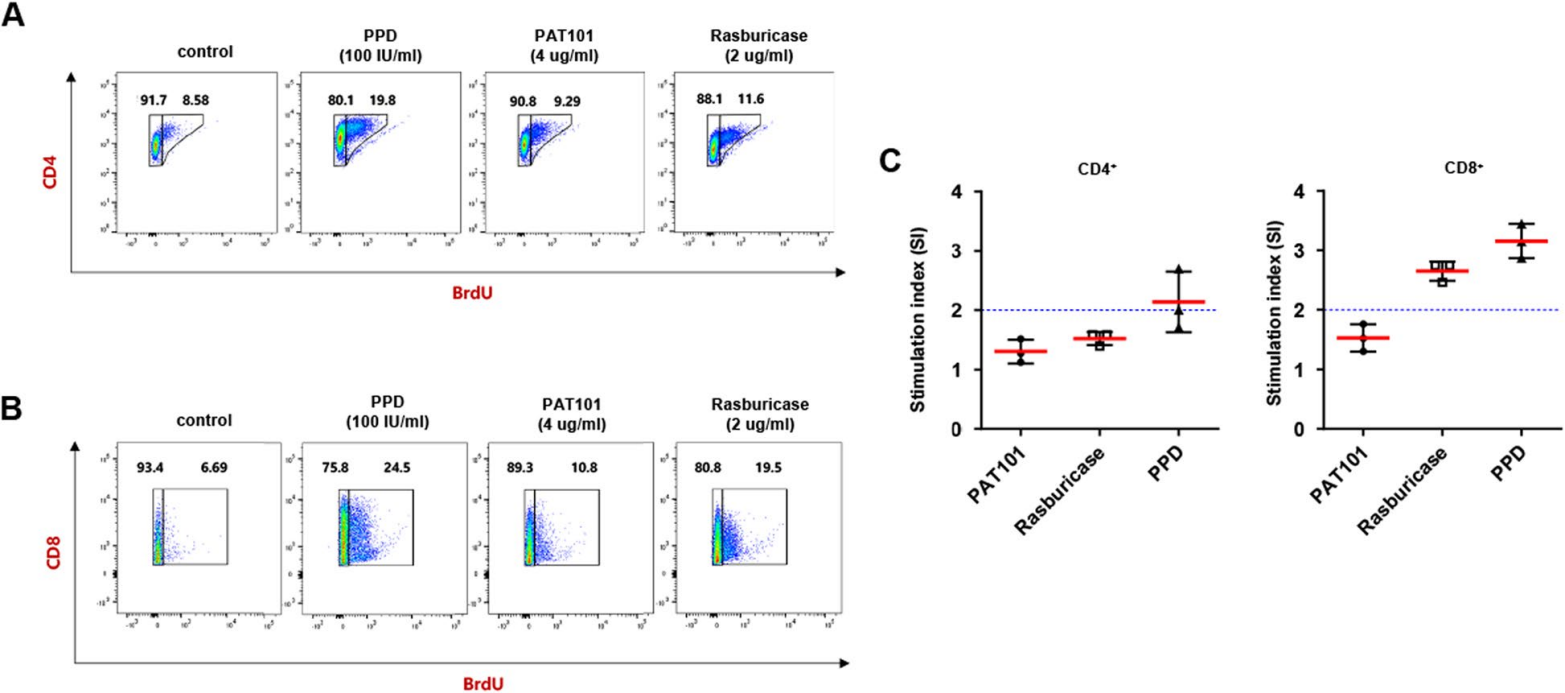
The immunogenic responses of CD4^+^T/CD8^+^T cell to PAT101 or rasburicase. **A**, **B** Increase in the positive population of CD4^+^T-cell (**A**) and CD8^+^T cell (**B**) proliferative response (BrdU uptake) to PPD, PAT101, or rasburicase. **C**, **D** Stimulation index (SI) of CD4^+^T-cell (**C**) and CD8^+^T cell (**D**) to PAT101, rasburicase, and PPD were plotted. The blue dotted line indicates that the SI value is 2, and cases with SI ≥ 2 were considered positive. Purified protein derivative (PPD) was used as a positive control. Data is presented as mean ± SD

The human PBMC-based assay was performed to predict immunogenicity risk response to PAT101 by quantifying the in vitro CD4^+^ T cell and CD8^+^ T cell, the critical indicators of immunogenicity. The positive immunological response to the PPD (Purified Protein Derivative) antigen will be the control for immunogenicity assessment. As shown in Fig. 5A and B, proliferative CD4^+^ T/CD8^+^ T cell responses to 100 IU/mL PPD, 4 μg/mL PAT101, and 2 μg/mL rasburicase were analyzed. The responses to PAT101 and rasburicase were lower than that of PPD in CD4^+^ and CD8^+^ T cells, and above all, the response to PAT101 was lower than that of rasburicase. The stimulation index (SI) is a measure for judging whether the response to the tested chemical is positive or negative. In this study, a positive response is defined as SI > 2 for a given compound [24]. The mean (SI) values of CD4^+^ T/CD8^+^ T cells by PAT101 and rasburicase were 1.30/1.52 and 1.51/2.65, respectively (Fig. 5C). SI to rasburicase was lower than the normal range of 2 in CD4^+^ T cells (Fig. 5A and C) but higher than 2 in CD8^+^ T cells (Fig. 5B and C), while SI to PAT101 were lower than 2 in both CD4^+^ T/CD8^+^ T cells (Fig. 5A–C). In conclusion, Fasturtec® was specifically positive for CD8^+^ T cells, whereas PAT101 was clearly negative for immunogenicity.

## Discussion

The prevalence of gout has been increasing over the last decades according to the National Health and Nutrition Examination Survey (NHANES). This increase is likely due to an aging population, changes in eating and lifestyle, rising rates of obesity, and increased use of drugs such as diuretics, all of which can increase uric acid levels in the body [26, 27]. Inhibiting uric acid synthesis and reabsorption as well as promoting uric acid hydrolysis can be an alternative to effectively manage hyperuricemia and gout. Uric acid-lowering drugs can be largely divided into three types [28, 29]. Xanthine oxidase inhibitors, classified as purine analogs (e.g., allopurinol) and non-purine analogs (e.g., febuxostat), can lower endogenous uric acid production and further lower uric acid levels [30]. Uric acid transporter 1 (URAT1) inhibiters including probenecid and lesinurad restrain the tubular urate reabsorption, facilitate urinary uric acid excretion, and reduce serum urate concentrations [31, 32]. As an enzyme therapy, exogenously applied Uox including rasbricase (Fasturtec®) and pegloticase (Krystexxa®) metabolizes uric acid into a soluble form, allantoin [33]. In chronic refractory gout, where traditional xanthine oxidase inhibitors are not available, recombinant Uox has emerged as a promising treatment option, showing rapid reduction of hyperuricemia and dramatic tophi regression [33]. Although these available recombinant urate oxidases are potent hypouricemic agents for chronic gout, their long-term use remains questionable. Rasburicase has been reported to have a short half-life of about 21 hr and to show immunogenic responses like repeated gout flares and hypersensitivity reactions in chronic gout [33]. The mean half-life of pegloticase is approximately 2 weeks through hyper-PEGylation, but many patients form anti-drug antibodies (ADAs), which are associated with loss of urate-lowering efficacy. There is also a subsequent risk of allergic reactions [33, 34]. The mechanism by which the human immune system induces an anti-PEG response to PEGylated proteins has not been clearly identified and is somewhat controversial in the literature, but it is still difficult to completely rule out the possibility of immunogenicity.

Site-specific rHA conjugation to therapeutic proteins through biorthogonal chemistry has the potential to extend the serum half-life of therapeutic proteins without compromising its activity. We have developed PAT101, one of the rHA-conjugated Uox variant candidates with comparable enzymatic activity and thermostability to those of *Af*Uox-WT [20]. In this study, PK profiles and efficacy of PAT101 were investigated to determine if PAT101 is a potential new therapeutic option for chronic gout patients. In particular, the PK profiles in TG mice clearly demonstrated that the half-life of PAT101 was twice that of pegloticase after a single dose (Fig. 2). In pegloticase, the mechanism of increased half-life is through the evasion of renal filtration due to volumetric increase by PEGylation. Compared to general mice (Fig. 1), the half-life increased more than twice in TG mice administered with only PAT101, not pegloticase (Fig. 2), confirming that PAT101 increases half-life not only by volume increase but also by FcRn mediated recycling. In SD rats repeatedly administered with PAT101 for 4 weeks, the activity of rasburicase decreased to 24%, while that was maintained up to 86%. Repeated administration reduced the activity in rasburicase due to ADA inevitably being produced by the Uox itself, but it is predicted that Uox surrounded by albumin, PAT101, can maintain its activity despite the production of ADA. Before the clinical trial, a PK study in NHP was performed to predict the PK profile of PAT101 in humans. The inclusion of NHP models in PK studies enhances understanding of drug behavior and holds the potential to streamline drug development by providing more accurate predictions in humans. After linear trapezoidal calculation, the half-life and AUC of PAT101 were 107.9 hr and 21,490.0 hr*mU/mL, respectively (Fig. S1A). In addition, binding affinity using surface plasmon resonance (SPR) between PAT101 and human-FcRn or monkey-FcRn were analyzed. The KDs (equilibrium dissociation constant) measured between PAT101 and human-FcRn or monkey-FcRn were 2.94 µM and 6.85 µM, respectively, demonstrating that the affinity of PAT101 with human-FcRn was about 2.3-fold higher than that with monkey-FcRn. The exceptionally long serum half-life of human serum albumin (approximately 21 days) is due to FcRn-mediated recycling [15–18, 35]. PAT101 is a rHA-conjugated Uox that is expected to trigger FcRn-mediated recycling and increase its half-life in vivo. Therefore, by confirming the difference in affinity with PAT101 between the two species, it will be possible to estimate the half-life of PAT101 in humans. It was predicted to be approximately 248.2 hr by linear calculations based on the amount of drug administered to humans using Human Equivalent Doses (HED) calculation and the FcRn binding capacity (Fig. S1B and C). Uox KO mice model somewhat mimics the disease of patients with severe hyperuricemia and renal impairment. This model has shown that PAT101 administration significantly increased the survival rate of Uox KO mice, which was comparable to that of pegloticase (Fig. 4C).

Considering the engineered Uox that has been marketed so far, a significant reduction in immunogenicity is a key objective in the development of a new Uox. As such, in human PBMC-based CD4^+^/CD8^+^ T-cell activation analysis, rasburicase was specifically positive for CD8^+^ T cells, whereas PAT101 was clearly negative for immunogenicity (Fig. 5). This positive response in rasburicase indicates that the recombinant Uox protein derived from *Aspergillus flavus* is a completely foreign body to the human immune system, which can induce T-cell immunogenicity. However, in the case of rHA-conjugated PAT101, this immune response seems to be alleviated, demonstrating that site-specific rHA conjugation to the therapeutic proteins mitigates immunogenic risk, even if it is not of human origin. Numerous biological products have been approved by the FDA. A review of these products found that immunogenicity was reported in 89% of cases, with half of these incidences affecting the efficacy of the drug [36, 37]. The immunogenicity of biopharmaceuticals is assessed by detecting and measuring antibodies or ADAs generated against these drugs. Multiple approaches (i.e., in silico prediction, in vitro cellular assays, and in vivo testing of ADAs from blood samples of nonclinical species and human clinical trials) have been considered to be applied during drug development, preclinical and clinical phases, and post-marketing drug surveillance [25, 38]. Preclinical immunogenicity assessment is vital during biopharmaceutical development. FDA guidance mentions that immunogenicity in animal models is not predictive of immunogenicity in humans, a poor correlation between animal models and clinical trials, due to species differences [39]. Consequently, in vitro assays that assess T cell responses from healthy donors to specific biopharmaceuticals is still recommended as a sensitive approach to predict immunogenicity in humans [39]. Nevertheless, the predictive value based on the relationship between in vitro T cell assays and clinical immunogenicity is still considered low. Therefore, we are considering the possibility that PAT101 may be positive for immunogenicity in clinical trials, unlike the in vitro results.

All the results such as half-life extension of Uox, uric acid reducing efficacy and lower risk of immunogenicity indicate that PAT101, a rHA conjugated *Af*Uox, could be a promising alternative as a Uox enzyme therapy for the treatment of severe and refractory gout. After biorthogonal reactions for Uox and rHA conjugation, the conjugates originally consist of the mixture of rHA-conjugated Uox variants including Uox-rHA1 (monomer), Uox-rHA2 (dimer), Uox-rHA3 (trimer) and Uox-rHA4 (tetramer). We found that the half-life increased proportionally with the number of albumin molecule(s) bound to Uox, from monomer to trimer [20]. Regarding trimers and tetramers, regression analysis showed that there were no significant differences in activity between them. Therefore, PAT101 will be optimized with only trimers and tetramers through a process development, and eventually, the efficacy and half-life of PAT101 might be further improved.

## Conclusions

PAT101 is a site-specific rHA-conjugated Uox developed for the treatment of gout. The MoA of dramatic half-life extension of PAT101 is achieved through FcRn-mediated recycling as well as volume-induced renal filtration evasion. Results from In vivo studies demonstrated that PAT101 could be a promising treatment for gout that overcomes the problems from previous treatments.

### Supplementary Information

**Additional file 1: Fig.S1.** PK profile of PAT101 in monkey (A) and binding assay of PAT101 with monkey- or human-originated FcRn by Surface Plasmon Resonance (SPR) (B-C).

## Data Availability

All data that support the findings of this study are available from the corresponding author upon reasonable request. However, there are restrictions to the availability of PAT101 related to company secret.

## Abbreviations

rHA: recombinant Human Albumin
Uox: Urate oxidase
IEDDA: Inverse electron demand Diels–Alder
NNAA: Non-natural amino acid
SPAAC: Strain-promoted azide-alkyne cycloaddition
FcRn: Neonatal fragment crystallizable receptor
PK: Pharmacokinetic
PBMCs: Peripheral blood mononuclear cells
TG: Transgenic
NHANES: National Health and Nutrition Examination Survey
AUC: Area under the curve
MoA: Mechanism of action
