# Supplementary material for: In vivo study of newly developed albumin-conjugated urate oxidase for gout treatment
Source: Arthritis Res Ther. 2023 Dec 18;25:247. doi: 10.1186/s13075-023-03231-3 (PMC10726570; doi:10.1186/s13075-023-03231-3)
Supplement: Supplementary file 1 — Additional file 1: Fig.S1. PK profile of PAT101 in monkey (A) and binding assay of PAT101 with monkey- or human-originated FcRn by Surface Plasmon Resonance (SPR) (B-C). [file 13075_2023_3231_MOESM1_ESM.docx]

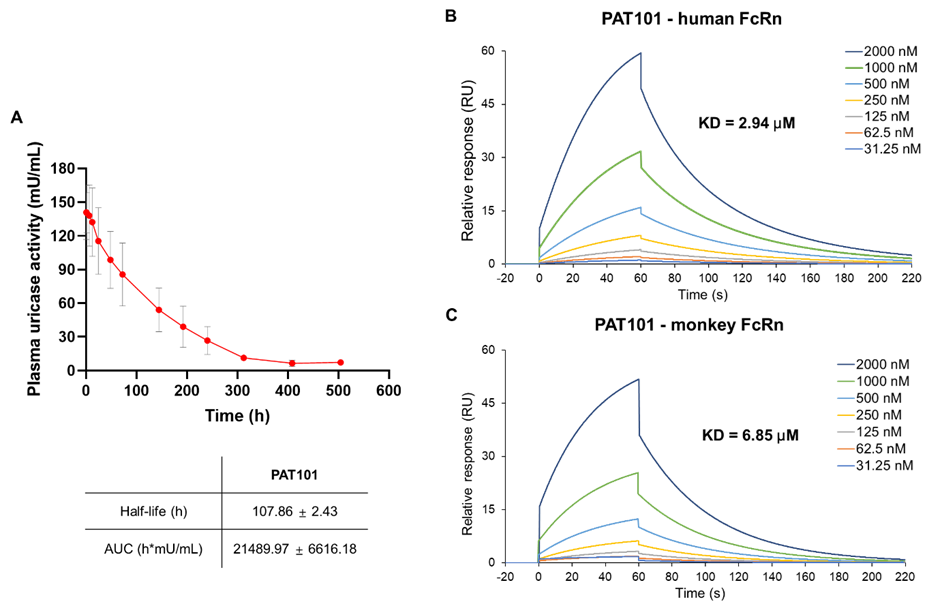


**Fig. S1** **PK profile of PAT101 in monkey (A) and binding assay of PAT101 with monkey- or human-originated FcRn by Surface Plasmon Resonance (SPR) (B-C)**

(**A**) Cynomolgus Monkeys (*Macaca fascicularis*) were purchased from Nafovanny (Tam Phuoc Hamlet, Bien Hoa City, Dong Nai Province, Vietnam). The Institutional Animal Care and Use Committee (IACUC) at the Safety Evaluation Institute of KIT has granted approval for the deliberation (KIT-N223004). On the day of administration, PAT101 (1.5 mg/kg) was intravenously administered to the monkeys while securely positioned in the monkey chair. The administration was carried out as a bolus injection. After administration for all monkeys, blood collection was executed from the lateral cortical vein at scheduled time points (0.5, 1, 6, 12, 24, 48, 72, 144, 192, 240, 312, 360, 408, and 504 hr). A total of 1.0 mL of blood was collected and transferred into collection tubes containing coagulant (EDTA-2K). Following gentle agitation of the collected blood, plasma was isolated via centrifugation at 3,000 rpm for 10 minutes at 4°C. The pharmacokinetic parameters were evaluated with isolated plasma using the identical methodology as described in the **Methods.**

(**B-C**) The Series S Sensor Chip CM4 (Cytiva, Uppsala, Sweden) was activated for 420 s with the mixture at a flow rate of 10 µL/min. 5 µg/mL of human- or monkey-FcRn in immobilization buffer (10 mM Sodium Acetate pH4.5) was injected to Biacore 8K channel (Cytiva, Uppsala, Sweden) at a flow rate of 10 µL/min. The chips were deactivated by 1 M Ethanolamine hydrochloride-NaOH at a flow rate of 10 µL/min for 420s. PAT 101 diluted in a range of concentrations (31.25-2,000 nM) was injected to channel at a flow rate of 30 µL/min an association and dissociation phase of corresponding time. The association and dissociation process were conducted in running buffer (PBS, pH6.0, with 0.05% Tween 20). The determination of binding affinity was calculated using Biacore Insight Evaluation software (Cytiva, Uppsala, Sweden).
